# Detection and Characterization of Human Enteroviruses, Human Cosaviruses, and a New Human Parechovirus Type in Healthy Individuals in Osun State, Nigeria, 2016/2017

**DOI:** 10.3390/v11111037

**Published:** 2019-11-07

**Authors:** Folakemi Abiodun Osundare, Oladele Oluyinka Opaleye, Akeem Abiodun Akindele, Samuel Adeyinka Adedokun, Olusola Anuoluwapo Akanbi, Claus-Thomas Bock, Sabine Diedrich, Sindy Böttcher

**Affiliations:** 1 Department of Medical Microbiology and Parasitology, Ladoke Akintola University of Technology, Osogbo 230222, Nigeria; 777folasegun@gmail.com (F.A.O.); yopaleye@yahoo.com (O.O.O.); aaakindele@lautech.edu.ng (A.A.A.); saadedokun27@lautech.edu.ng (S.A.A.); AkanbiO@rki.de (O.A.A.); 2 Science Laboratory Technology Department, Federal Polytechnic, Ede 232101, Nigeria; 3 Department of Infectious Diseases, Viral Gastroenteritis and Hepatitis Pathogens and Enteroviruses, Robert Koch Institute, 13353 Berlin, Germany; 4 Institute of Tropical Medicine, University of Tuebingen, 72074 Tuebingen, Germany; 5 National Reference Center for Polioviruses and Enteroviruses, Robert Koch Institute, 13353 Berlin, Germany; DiedrichS@rki.de

**Keywords:** enteroviruses, parechoviruses, cosaviruses, poliovirus, containment, Nigeria, picornavirus

## Abstract

Human enteroviruses and human parechoviruses are associated with a broad range of diseases and even severe and fatal conditions. For human cosaviruses, the etiological role is yet unknown. Little is known about the circulation of non-polio enteroviruses, human parechoviruses, and human cosaviruses in Nigeria. A total of 113 stool samples were collected from healthy individuals in Osun State between February 2016 and May 2017. RT-PCR assays targeting the 5′ non-coding region (5′ -NCR) were used to screen for human enteroviruses, human parechoviruses, and human cosaviruses. For human enteroviruses, species-specific RT-PCR assays targeting the VP1 regions were used for molecular typing. Inoculation was carried out on RD-A, CaCo-2, HEp-2C, and L20B cell lines to compare molecular and virological assays. Ten samples tested positive for enterovirus RNA with 11 strains detected, including CV-A13 (*n* = 3), E-18 (*n* = 2), CV-A20 (*n* = 1), CV-A24 (*n* = 1), EV-C99 (*n* = 1), and EV-C116 (*n* = 2). Three samples tested positive for human parechovirus RNA, and full genome sequencing on two samples allowed assignment to a new Parechovirus A type (HPeV-19). Thirty-three samples tested positive for cosavirus with assignment to species Cosavirus D and Cosavirus A based on the 5′-NCR region. Screening of stool samples collected from healthy individuals in Nigeria in 2016 and 2017 revealed a high diversity of circulating human enteroviruses, human parechoviruses, and human cosaviruses. Molecular assays for genotyping showed substantial benefits compared with those of cell-culture assays.

## 1. Introduction

Human enteroviruses and human parechoviruses circulate worldwide, and transmission occurs mainly via the faecal-oral route and respiratory route [1,2]. They have been associated with a broad range of diseases, including mild gastrointestinal and respiratory symptoms as well as neurological disorders and even life-threatening conditions like neonatal sepsis, myocarditis, and acute flaccid paralysis [3]. Cosaviruses were first detected in stool samples of patients presenting acute flaccid paralysis and their healthy contacts [4,5]. They were also detected in patients with diarrhea in the work of da Costa et al. [6], but the etiological role of cosaviruses remains unclear to date [7].

The majority of both human enterovirus and parechovirus infections are asymptomatic; several studies have described detection of human enteroviruses and human parechoviruses in healthy individuals, including two reports from Sub-Saharan Africa [8,9]. Recently, a few studies have shed more light on the presence and circulation of human enterovirus types in healthy and symptomatic individuals in Nigeria. The studies were carried out in the framework of the Global Polio Eradication Initiative and therefore focused on children below the age of 15 years [10,11,12,13,14]. For circulation of human parechoviruses in Nigeria, no information is available to date. However, Cosavirus A strains and Cosavirus E/D strains (recombinant strain) were reported in the stool samples of acute flaccid paralysis (AFP) patients in Nigeria [15]. Surveillance is pivotal to detecting and understanding the biology and impact of these viruses.

In this report, we present the results of stool samples collected from healthy Nigerian individuals (children and adults) during a 16-month period and tested for human enteroviruses, human parechoviruses, and cosaviruses. Positive samples were characterized using molecular assays. For enterovirus strains detected by molecular methods, cell culture assays were additionally used.

## 2. Materials and Methods 

A total of 113 stool samples were collected from apparently healthy children and adults in Osun state, Nigeria for the purpose of a Hepatitis E study and to identify other viruses present in healthy individuals in Nigeria. The samples were collected from 56 females (49.6%) and 57 males (50.4%). The samples were collected from the Oke-Osun, Ede, and Ore communities, all of which are in Osun State. Informed verbal consent was obtained from volunteers and the parents/guardians of children. The samples were collected in February 2016 (Ede), March 2016 (Oke-Osun), July 2016 (Ede), September 2016 (Ede), and May 2017 (Ore). They were stored at −80 °C until transport to the Robert Koch Institute in Berlin, Germany for analysis. The exclusion of poliovirus allowed handling of these samples outside of polio containment conditions in the context of the WHO Global Action Plan III [16].

Suspensions of fecal specimens from humans were prepared by vortexing 0.1 g of feces with 1 mL of PBS. The suspensions were clarified at 10,000 rpm for 8 min at 25 °C. 

RNA extraction was carried out using the QiaCube according to the protocol recommended by the manufacturer, using a 135 µL stool suspension or cell culture supernatant. As an internal control, 5 µL containing a defined number of MS2 phage particles were added. The extraction products were collected and stored at −80 °C before use.

Screening for human enteroviruses, human parechoviruses, and cosaviruses was carried out using one-step RT-PCR assays followed by nested PCR targeting the corresponding 5′ non-coding region (5′-NCR). These allowed sequencing of the PCR amplicon and subsequent assignment to enterovirus, cosavirus, and parechovirus species [4,17,18]. Molecular typing of enteroviruses was carried out using a pan-enterovirus assay [19] and species-specific primer systems: Enterovirus A [20], Enterovirus B [21], Enterovirus C [this study, supplementary material, and [22], and Enterovirus D [23]. Parechovirus typing was done using primers targeting the VP1 region [8]. Sequencing was carried out on PCR products using BigDye 3.1 (Applied Biosystems, Foster, MA, USA ). Consensus sequences were assembled using the Sequencher software (www.genecodes.com, Ann Arbor, MI, USA). Enterovirus Genotyping Tool [24] and NCBI BLAST [25] were used to assign enterovirus and parechovirus types as well as cosavirus species. Nearly full genome sequencing of two Parechovirus A strains is described in the supplementary material. Enterovirus VP1 region sequences were submitted to GenBank under accession numbers MK531846-MK531855. Parechovirus genome sequences were submitted to GenBank under accession numbers MN307882 and MN307883.

Alignments of strains identified in this study and representative reference strains from Genbank^®^ were performed using the MAFFT algorithm implemented in Geneious 11.1.5 (Biomatters Ltd, Auckland, New Zealand. The Maximum Likelihood and Neighbor-Joining trees were calculated using MEGA 7.0.26 under a GTR+G+I evolution model. Only bootstrap values >70 are shown [26].

Cell culture for virus isolation was performed as recently described [27]. Presence of cytopathic effect (CPE) was checked daily; passaging supernatant to fresh cells was performed after 5 (HEp-2C) or 7 (RD-A, CaCo-2, L20B) days. Up to four passages were conducted, due to the high toxicity of most of the stool suspensions on the inoculated cell lines. Supernatants were tested with the enterovirus 5′-NCR nested-RT-PCR assay. All supernatants that were enterovirus positive were then tested by an Enterovirus real-time PCR kit (Argene^®^, Biomerieux, Marcy-l’Étoile, France) to compare quantitative genomic content (Cp values) of the stool samples vs. supernatant.

## 3. Results

**Figure 1:**
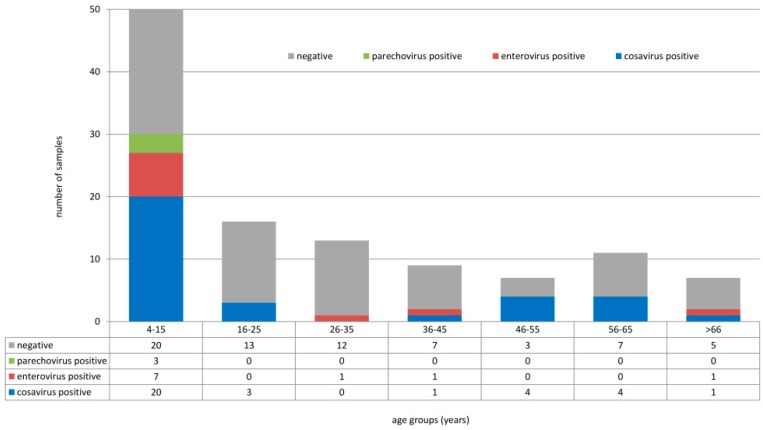
Detection of human enteroviruses, human parechoviruses, and cosaviruses in different age groups.

Overall, 113 stool samples of healthy individuals (children and adults ranging from 4 to 84 years of age) were collected in three different areas in the southwestern part of Nigeria between February 2016 and May 2017. Screening for human enteroviruses, human parechoviruses, and cosaviruses revealed that ten samples tested positive for enteroviruses, three tested positive for human parechoviruses, and 33 tested positive for cosaviruses. Human enteroviruses were detected in age groups 4–15 years (7/50), 26–35 years (1/13), and >66 years (1/7). Human parechoviruses were detected in children only (4–15 years, 3/50). Cosaviruses were detected in all age groups: 4–15 years (20/50), 16–25 years (3/16), 36–45 years (1/9), 46–55 years (4/7), 56–65 year (4/11), and >66 years (1/7), as seen in Figure 1. Human enteroviruses were detected in all three locations: Ore (6/28), Oke-Osun (2/58), and Ede (2/27), as seen in Table 1. Human parechovirus detections occurred only in Ore, as seen in Table 2 and Table 3. Cosaviruses were also detected in all three regions: Ore (15/28), Oke-Osun (14/58), and Ede (4/27) (Table 3, File S1).

**Figure 2:**
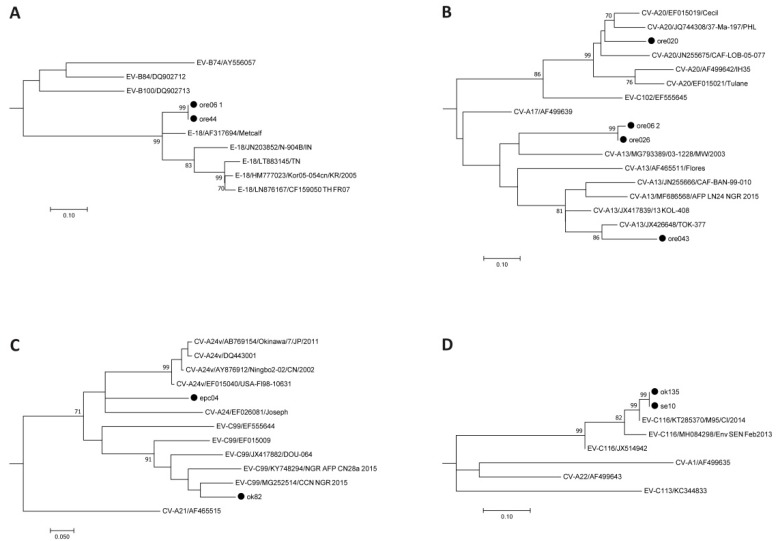
Enterovirus type assignment based on the VP1 region. The evolutionary history was inferred using the Neighbor-Joining method and evolutionary distances were calculated using the Maximum Composite Likelihood model; rate variation among sites was modeled by Gamma distribution. Reference sequences available in Genbank were used. Subtrees for Echovirus 18 (**A**), Coxsackievirus A13 and A20 (**B**), Enterovirus C99 and Coxsackievirus A24 (**C**), and Enterovirus C116 (**D**) are shown. Strains identified within this study are marked with a filled circle. The complete tree is available in the File S1.

Ten samples tested positive for human enteroviruses. Sequencing of the 5′-NCR region revealed two Enterovirus B and eight Enterovirus C strains. Based on the VP1 region sequence, strains could be assigned to enterovirus types Echovirus 18 (E-18, *n* = 2), Coxsackievirus A13 (CV-A13, *n* = 3), CV-A20 (*n* = 1), CV-A24 (*n* = 1), Enterovirus C99 (EV-C99, *n* = 1), and EV-C116 (*n* = 2)—as seen in Table 2—using BLASTn, the Enterovirus Genotyping Tool, and phylogenetic tree calculation, as seen in Figure 2. The presence of two enterovirus strains in the sample ore 06 was solved by using species-specific PCR assays. No poliovirus sequences were obtained by using the poliovirus-specific PCR assay. One strain remained negative in the VP1 assays (ore 047) but the presence of poliovirus was excluded by a cell culture assay.

Four different cell lines (RD-A, CaCo-2, HEp-2C, and L20B) were inoculated with human enterovirus—as seen in Table 2—and human parechovirus-positive stool suspensions. Only HEp-2C cell lines supported the growth of the two CV-A13 strains (ore 06, ore 026) as well as CV-A20 (ore 20) and CV-A24 (epc 04). No CPE was detected on RD-A and CaCo-2 cells, but for one sample (ore 026), real-time PCR of the cell culture supernatant resulted in a higher Cp value than was detected in the original stool sample, indicating non-lytic replication of CV-A13. Notably, we recognized non-enterovirus CPE in the third passage on L20B, which disappeared after repeatedly passaging to fresh cells. Only weak Cp values for enterovirus RNA could be detected in several cell culture supernatants (epc 04, se 10, ok 82, ore 06, ore 44), which were most probably diluted leftover genomes contained in the stool samples; no increase in enterovirus RNA was detected in the remaining supernatants. Neither of the recently detected enterovirus types (EV-C99 and EV-C116) could be isolated. In addition, none of the parechovirus-positive samples resulted in CPE in any of the four cell lines 

Three samples which tested positive for human parechoviruses were identified (ore 33, ore 44, ore 39). All three were collected in May 2017 and originated from children of 5, 8, and 10 years of age, as seen in Table 2 and Table 3.

**Figure 3:**
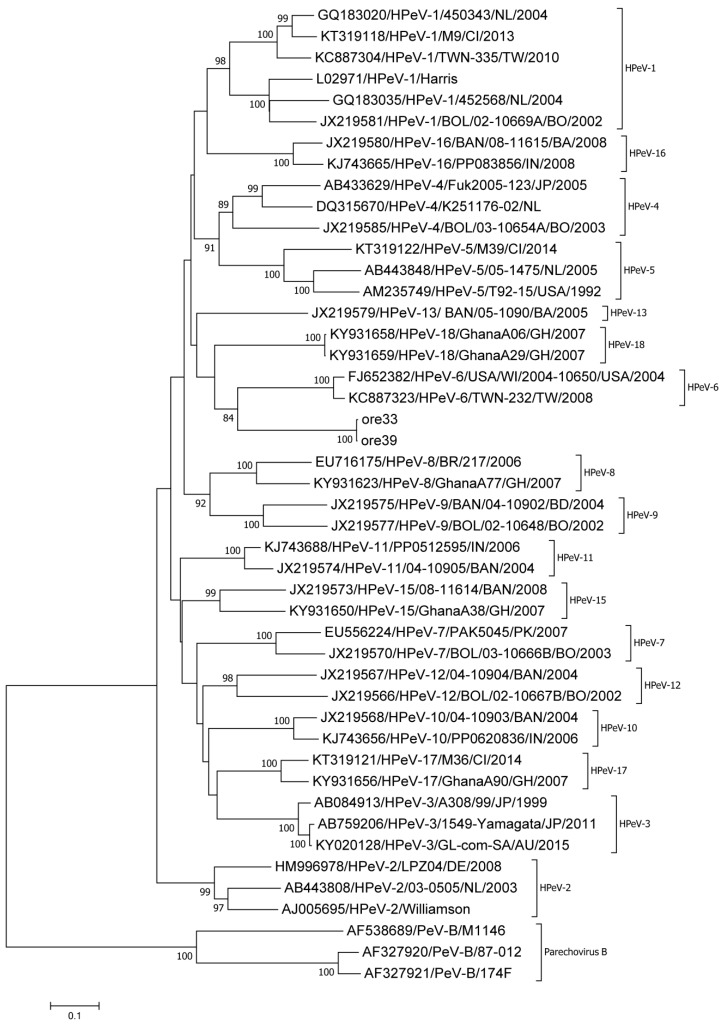
Phylogenetic assignment of two Parechovirus A strains identified within this study, ore 33 and ore 39, based on the complete VP1 region. The evolutionary history was inferred using the Neighbor-Joining method. Evolutionary distances were calculated using the Maximum Composite Likelihood model and rate variation among sites was modeled by Gamma distribution. Forty reference sequences available in Genbank were used, including prototypes and current circulating strains of the species Parechovirus A as well as Parechovirus B strains as outgroup.

The VP1 region could be amplified for two samples (ore 33, ore 39). Both sequences showed only two nucleotide differences with one another. Using BLAST, an approximately 75% nucleotide identity to HPeV-6 (FJ373178) was identified, which was close to the known cutoff values for enterovirus [28] and parechovirus [29] types. Using the Enterovirus Genotyping Tool, a low bootstrap support of 70 was found. In addition, the two sequences clustered separately in a phylogenetic tree based on the VP1 region, as seen in Figure 3.

Due to the low nucleotide identities between the VP1 regions of the two Parechovirus A strains and the reference strains, we assembled nearly the entire genome (ore 33, ore 39) by next-generation sequencing of two overlapping PCR fragments (see supplementary material). The 3′-NCR was retrieved through 3′ RACE. For both strains, a 7021 nt consensus sequence representing the 381 nt covering the 5′-NCR, P1 region (2316 nt), P2 region (1800 nt), P3 region (2421 nt), and 87 nt 3′-NCR was obtained. Both sequences were deposited in Genbank under accession numbers MN307882 and MN307883. Both strains showed a high nucleotide identity with only 10 positions differing in the entire genome sequence. Phylogenetic analysis of the VP1 region showed clear separation of these two strains, as seen in Figure 3. When compared with HPeV-1 to -8 and HPeV-17, nucleotide and amino acid identities of the P1 region did not exceed 71% and 81%, respectively, suggesting that these two strains represent a new Parechovirus A genotype. We sent an assignment request to the Picornavirus study group; the strains were classified as a new Parechovirus A, HPeV-19 (Roland Zell, personal communication).

Thirty-three samples tested positive for cosaviruses. Assignment of cosavirus species was done through amplification of a 236 bp fragment of the 5′-NCR and submission of the sequence to the Enterovirus Typing Tool [24]. Of the 33 samples that were positive for cosavirus, nine were assigned to the species Cosavirus A (27.3%) and 24 to Cosavirus D (72.7%) (Table 3, File S1). Three of the cosavirus-positive samples also tested positive for enteroviruses (ore 20, ore 43, and ore 44); we therefore tested the cell culture supernatants for cosaviruses. Positive PCR results were obtained for two supernatants of ore 43 and ore 44. Since we have not established a cosavirus qRT-PCR, we cannot exclude genome leftovers detected by the nested PCR assay and further investigation is needed to clarify detection of cosavirus in cell culture supernatants.

One sample showed co-infection with a human enterovirus, a human parechovirus, and a cosavirus (ore 44). This highlights the benefits of specific molecular assays compared to cell culture, which is usually undirected and—besides the laboratory capacities—depends on the presence of infective particles and receptor compatibility.

## 4. Discussion

Nigeria is a country in which polio is still endemic, and focus on the eradication of polio through a concerted effort in AFP surveillance has been heightened. However, there is a dearth of information on the prevalence of the non-polio enteroviruses (NPEV)—particularly Enterovirus C—as well as the prevalence of human parechoviruses and the newly described cosaviruses. Although most infections caused by NPEV and human parechoviruses are often asymptomatic, they sometimes result in severe and fatal outcomes. Human parechoviruses have also been associated with severe infections, especially in neonates. Little is known of the etiological role of cosaviruses.

In the study presented here, enterovirus RNA was detected in 10/113 (8.8%) stool samples obtained from apparently healthy individuals between 4 and 84 years of age. Six different enterovirus types could be identified among 11 strains, of which nine belonged to Enterovirus C. This is in line with the results from other studies in neighboring countries [8,9,30]. Members of the species Enterovirus C have been identified to be more prevalent in apparently healthy individuals [14,15,31]. Some of the enterovirus strains isolated in this study have been associated with different diseases from previous reports. EV-C116 has been associated with diarrhea in children [32] while EV-C99 has been associated with acute flaccid paralysis [33]. CV-A24 has been recently associated with conjunctivitis [30], and Echovirus 18 has been documented in outbreaks of aseptic meningitis worldwide, e.g., Germany [34] and China [35]. Amongst others, CV-A13 and CV-A24 have been detected in non-human primates [36], suggesting a zoonotic potential of these enterovirus strains. 

Great success has been achieved by the Global Polio Eradication Initiative (GPEI), with only three countries still endemic for wildtype poliovirus Type 1 (WPV-1). In addition to Pakistan and Afghanistan, Nigeria was one of these countries. In 2016, a WPV-1 outbreak occurred in the northern part of Nigeria (Borno state) and in addition, circulating vaccine-derived polioviruses type 2 (cVDPV-2) were detected. Recently published data showed high recombination frequency of polioviruses with NPEV EV-C strains [31,37]. A poor sanitation practice that fosters the spread of non-polio enteroviruses and low herd immunity to polio might be a fertile ground for the emergence of these recombinant cVDPV/NPEV group C strains. This recombination could lead to strains with a boomerang effect of wild-type pathogenicity and transmissibility, and should be monitored to immediately stop transmission chains [13,38,39,40,41].

Reports on non-polio enterovirus typing from Nigeria have previously been biased by using cell culture methods only, showing a predominance of strains belonging to Enterovirus B [10,11,12]. However, with the use of molecular techniques, Adeniji et al. [14] found that 64.3% of the stool samples collected from AFP patients tested positive for strains belonging to Enterovirus C. In our study, only 4 of the 11 enterovirus strains identified by PCR assays were detectable via cell culture assays. The inclusion of the HEp-2C cell line with the routine cell lines used for poliovirus isolation has been shown to increase the probability of isolating different non-polio enterovirus species C genotypes [31]. Nevertheless, the percentage yield from cell culture is quite inadequate and allows for the missing of some strains of non-polio enteroviruses. Adeniji et al. [14] showed that there was no isolation of enteroviruses using the RD-L20B algorithm but that a 50% yield was obtainable using molecular assays. In this study, we identified a double infection in one sample (ore 06) with molecular methods only. Additional isolation of CV-A13 from HEp-2C cells endorses the fact that molecular assays and cell culture techniques should be carried out in parallel for comprehensive enterovirus investigations.

This study also highlights the need for further investigation of the circulation of human parechoviruses in Nigeria. The relatively low number of Parechovirus A strains identified in comparison with other studies might be explained by the different age distribution of our cohort, where the individuals were aged 4 years and older. The Parechovirus A strains were found in two males and one female of 10, 8, and 5 years of age. This is in congruence with the findings of di Cristanziano et al. [8], who reported the presence of Parechovirus A infections in older children. In contrast, severe HPeV-3 infections have been reported mainly in infants below the age of 3 months [42]. Near-full genome sequencing revealed a new Parechovirus A type, with the confirmatory assignment provided by the picornavirus study group. Comparing nucleotide differences of the P1 region showed no more than 71% nucleotide identities and 81% amino acid identities with other known parechovirus types; both were far below the suggested cutoff for the parechovirus VP1 region [29]. All three parechovirus strains in this study were identified in the Ore region, probably explaining the high nucleotide identities. Further studies are needed to better understand the impact and epidemiology of human parechovirus strains circulating in Nigeria.

The high detection rate of cosaviruses in nearly all age groups of our cohort indicates widespread circulation of these viruses. Since cosaviruses have been detected in both healthy and symptomatic individuals and case-cohort studies are still missing, the etiological role remains unclear. To determine whether cosaviruses are the causative agents of neurological or gastrointestinal disorders or act as bystanders needs further investigation [43,44].

Furthermore, three individuals showed co-infection with an enterovirus and a cosavirus, and one individual was infected with an enterovirus, a parechovirus, and a cosavirus. However, all samples were collected from healthy children, indicating not only a widespread and high number of viruses circulating among individuals in this age group, but also suggesting co-factors needed for these viruses to establish a symptomatic infection. A recent study showed a high number of dual infections among children participating in regular testing for enteroviruses and adenoviruses [45].

In summary, non-polio enteroviruses, human parechoviruses, and cosaviruses are circulating in Nigeria and could be easily detected by molecular assays. To the best of our knowledge, this is the first report of human parechoviruses in Nigeria, including the detection of a new parechovirus type. Syndromic surveillances should be carried out to understand the burden caused by these viruses in the population.

## Figures and Tables

**Table 1:** Characteristics of patients with enterovirus-positive samples and molecular results.

Sample ID	Name of Location	Type of Location	Age (Y)	Sex	Collection Date	5’-NCR seq	VP1 seq
epc 04	Ede	Urban	28	Male	September 2016	Enterovirus C	CV-A24
se 10	Ede	Urban	45	Male	September 2016	Enterovirus C	EV-C116
ok 82	Oke-Osun	Semi-Urban	4	Male	September 2016	Enterovirus C	EV-C99
ok 135	Oke-Osun	Semi-Urban	72	Female	September 2016	Enterovirus C	EV-C116
ore 06	Ore	Rural	5	Female	May 2017	Enterovirus B	E-18,CV-A13
ore 026	Ore	Rural	14	Male	May 2017	Enterovirus C	CV-A13
ore 043	Ore	Rural	4	Male	May 2017	Enterovirus C	CV-A13
ore 047	Ore	Rural	8	Female	May 2017	Enterovirus C	Negative
ore 20	Ore	Rural	11	Female	May 2017	Enterovirus C	CV-A20
ore 44	Ore	Rural	5	Female	May 2017	Enterovirus B	E-18

**Table 2:** Enterovirus isolation and enterovirus realtime RT-PCR results of stool samples compared to cell culture supernatants.

Sample ID	Sequencing Result	Cp Value Stool	CPE on RD-A	Cp Value RD-A Supernatant	CPE on CaCo-2	Cp Value CaCo-2, Supernatant	CPE on Hep-2C	CP Value Hep-2C, Supernatant	CPE on L20B	Cp Value L20B Supernatant
epc 04	CV-A24	29.15	negative	33.60	negative	38.46	positive	23.65	negative *	35.84
se 10	EV-C116	29.34	negative	36.77	negative	37.15	negative	36.01	negative *	32.22
ok 82	EV-C99	33.56	negative	40.0	negative	37.27	negative	n.d.	negative *	35.95
ok 135	EV-C116	40	negative	n.d.	negative	n.d.	negative	n.d.	negative	n.d.
ore 06	E-18, CV-A13	n.e.	negative	35.84	negative	37.67	positive	23.01	negative	n.d.
ore 026	CV-A13	33.11	negative	22.21	negative	n.d.	positive	24.95	negative	n.d.
ore 043	CV-A13	40	negative	n.d.	negative	n.d.	negative	n.d.	negative	n.d.
ore 047	Enterovirus C	38.78	negative	n.d.	negative	n.d.	negative	n.d.	negative *	n.d.
ore 20	CV-A20	28.81	negative	26.97	negative	26.76	positive	23.04	negative	n.d.
ore 44	E-18	25.06	negative	31.50	negative	26.50	negative	36.48	negative	n.d.

n.e. = not evaluable due to inhibition; n.d. = not detectable; * = no characteristic enterovirus CPE, reinoculation to RD-A cells remained negative.

**Table 3:** Characteristics of parechovirus positive patients and molecular results.

Sample ID	Name of Location	Location	Age (Years)	Sex	Collection Date	5’-NCR Seq	VP1 Seq
ore 44	Ore	Rural	5	female	May 2017	Parechovirus A	negative
ore 39	Ore	Rural	8	male	May 2017	Parechovirus A	unassigned
ore 33	Ore	Rural	10	male	May 2017	Parechovirus A	unassigned

## Supplementary Materials

The following are available online at https://www.mdpi.com/1999-4915/11/11/1037/s1, File S1.
