# Supplementary material for: Detection and Characterization of Human Enteroviruses, Human Cosaviruses, and a New Human Parechovirus Type in Healthy Individuals in Osun State, Nigeria, 2016/2017
Source: Viruses. 2019 Nov 7;11(11):1037. doi: 10.3390/v11111037 (PMC6893832; doi:10.3390/v11111037)
Supplement: Supplementary file 1 [file viruses-11-01037-s001.pdf]

Supplementary material

RT-PCR assay for typing enterovirus species C strains

Amplification of the VP3/VP1 junction region was performed using the One-Step-RT-PCR Kit (QIAGEN), followed by nested PCR using HotStarTaq-Mastermix (QIAGEN), according to the manufacturer's protocol. Reverse transcription PCR (RT-PCR) and nested PCR was done with 600 nM of forward and reverse primers as mentioned in Technical Appendix Table 1. The temperature profile for the VP1 region was the following: 45 min 45°C, 15 min 95°C for reverse transcription, followed by 10 touch-down cycles protocol of [30 s 94°C, 30 s 50°C, 90 s 72°C] with a decrease of 1°C of the annealing temperature per cycle, followed by 30 cycles of [30 s 94°C, 30 s 42°C, 90 s 72°C] and final elongation for 10 min at 72°C. The nested PCR was carried out by using a touchdown protocol with 10 cycles of [30 s 94°C, 30 s 52°C, 60 s 72°C, with a decrease of 1°C of the annealing temperature per cycle, followed by 30 cycles of [30s 94°C, 30 s 42°C, 60 s 72°C], and final elongation for 10 min at 72°C. The resulting PCR products of the nested PCR (785 bp) were directly sequence using primer NRZ 300 and NRZ 301.

Table 1: Primers used for EV-C VP1 assay.

Primer	Orientation	Sequence 5'-3'
NRZ 298	Sense	TAY TAY ACH CAY TGG RCN GGN TC
NRZ 299	Antisense	TGC CAN GTR TAR TCR TCC CA
NRZ 300	Sense	ATH TGG GAY VTN GGN YTN CAR TC
NRZ 301	Antisense	CCD GGD GGN AYR TAC ATD ATY TGR TA

Full-genome sequencing of the HPeV genome

Two nested RT-PCR assays for the amplification of the near-full HPeV genome was conducted using the PrimeScript One Step RT-PCR Kit Ver.2 (TaKaRa Bio Inc.). The two overlapping PCR fragments were amplified by primer combinations listed in Table 2. First-round RT-PCR assay were done using 5 µl RNA, 800 nM primers NRZ 127 and NRZ 214 for PCR-A and NRZ 212 and NRZ 429 for PCR-B, 1 µl PrimeScript 1 step Enzyme Mix, 12.5 µl 2X 1 step buffer, and 4.5 µl RNase free dH_2O. Reverse transcription was done at 50°C for 30 min followed by 94°C for 2 min. Amplification was done by 45 cycles at [94°C for 30 sec, 50°C for 30 sec, and 72°C for 300 sec]. Final elongation was one for 10 min at 72°C. Second-round semi-nested PCR used 800 nM primer NRZ129 and NRZ214 for PCR-A and NRZ 212 and NRZ 430 for PCR-B, 1 µl PrimeScript 1 step Enzyme Mix, 12.5 µl 2X 1 step buffer, and 7.5 µl RNase free dH_2O and 2 µl of the first-round RT-PCR product. Temperature profile was 2 min 94°C, followed by 45 cycles at [94°C for 30 sec, 50°C for 30 sec, and 72°C for 300 sec] and final elongation for 10 min at 72°C.

To amplify the very 3'end of the HPeV genome the One-Step-RT-PCR Kit (QIAGEN) and HotStarTaq-Mastermix (QIAGEN) according to the manufacturer's protocol. Reverse transcription PCR (RT-PCR) was done with 600 nM of primer NRZ 431 and 15T-aTag under following conditions: 30 min 50°C, 15 min 95°C followed by 35 cycles of [30 sec 94°C, 30 sec 55°C, 60 sec 72°C] and a final elongation of 72°C. Semi-nested PCR was done using primer NRZ 432 and 15T-aTag under following conditions: 15 min 95°C followed by [30 sec 94°C, 30 sec 60°C, 30 sec 72°C] and a final elongation for 10 min at 72°C. The resulting PCR product of 441 bp was directly sequenced using the ABI BigDye 3.1 kit with primers NRZ 431 and NRZ 432.

Supplementary Material Table 2: Primers used for amplification of the nearly full genome of the novel HPeV strain

Primer	Orientation	Sequence 5'-3'	Reference
NRZ 127	sense	GGGTGGCAGATGGCGTGCCATAA	253, Harvala et al., [1]
NRZ 214	antisense	TAGTGYTTGTARAAACCY CTATCTA	DiCristanziano,et al., [2]
NRZ 212	sense	GACAATAGTTTTGAAATNACWATMCC	DiCristanziano et al., [2]
NRZ 429	antisense	TCAAACACCATGGGCATCAAYTTAG	this study
NRZ 129	sense	YCACACAGCCATCCTCTAGTAAG	313, Harvala et al., [1]
NRZ 430	antisense	ACMACATCATAATCATCCAC	this study
NRZ 431	sense	CCATAYAAAGATTGGCACTTYATGATY AAT GC	this study
NRZ 432	sense	CCAGAGAAACTGCARAGTATCATGGCAGATTCATTTGG	this study
15T-aTag	antisense	GCCAACGACCGGGAGGCCAGCTTTTTTTTTTTTTTTV	Müller et al., [3]

The products of the semi-nested PCR assays (PCR-A = 2792 bp, PCR-B = 5123 bp) were purified using magnetic beads using MagSi-NGS^{PREP} Plus (Steinbrenner Laborsysteme GmbH, Wiesenbach, Germany). NexteraXT library preparation and Illumina sequencing were performed. DNA was quantified using the Qubit dsDNA HS Assay Kit (Life Technologies, Darmstadt, Germany) and shotgun DNA libraries were generated with the Nextera XT DNA Sample Preparation Kit (Illumina, San Diego, CA, USA). Library size was determined with High Sensitivity DNA Analysis Kits for the 2100 Bioanalyzer Instrument (Agilent Technologies, Waldbronn, Germany). Libraries were quantified using the KAPA Library Quantification Kit for Illumina (Kapa Biosystems, Wilmington, MA, USA) and pooled before sequencing. The library pool was sequenced on a MiSeq instrument (Illumina, San Diego, CA, USA) in a paired end sequencing run using the MiSeq Reagent Kit v3 600 cycle kit. Sequencing data underwent quality control using an in-house quality control pipeline based on Trimmomatic 0.32[26] and FastQC 0.11.2 (http://www.bioinformatics.babraham.ac.uk/projects/fastqc/). Trimmed reads were mapped to parechovirus strain AB252582 and JX826607 separately using the "Map to Reference" algorithm in Geneious version 11.1.5.

Supplementary Material Figure 1: Phylogenetic tree using the VP1 region of enterovirus strains detected in this study compared to reference strains of species EV-B and EV-C available in GenBank. The evolutionary history was inferred using the Maximum Likelihood method based on a GTR model. Enterovirus strains identified within this study are marked with filled circles. Subtrees are shown in Figure 2 in the manuscript text.

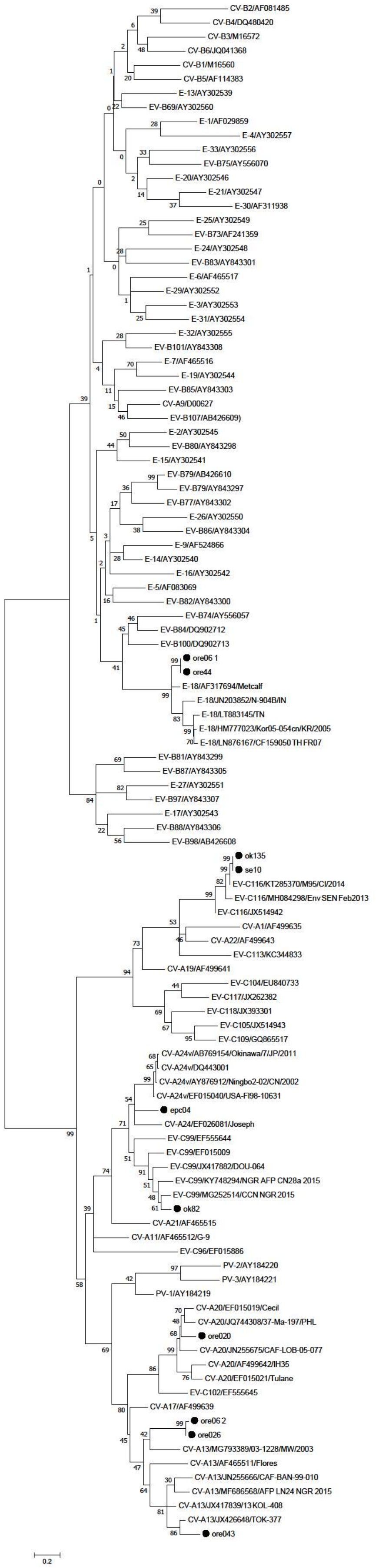

CV-B2/AF081485
CV-B4/DQ480420
CV-B3/M16572
CV-B6/JQ041368
CV-B1/M16560
CV-B5/AF114383
E-13/AY302539
EV-B69/AY302560
E-1/AF029859
E-4/AY302557
E-33/AY302556
EV-B75/AY556070
E-20/AY302546
E-21/AY302547
E-30/AF311938
E-25/AY302549
EV-B73/AF241359
E-24/AY302548
EV-B83/AY843301
E-6/AF465517
E-29/AY302552
E-3/AY302553
E-31/AY302554
E-32/AY302555
EV-B101/AY843308
E-7/AF465516
E-19/AY302544
EV-B85/AY843303
CV-A9/D00627
EV-B107/AB426609)
E-2/AY302545
EV-B80/AY843298
E-15/AY302541
EV-B79/AB426610
EV-B79/AY843297
EV-B77/AY843302
E-26/AY302550
EV-B86/AY843304
E-9/AF524866
E-14/AY302540
E-16/AY302542
E-5/AF083069
EV-B82/AY843300
EV-B74/AY556057
EV-B84/DQ902712
EV-B100/DQ902713
ore06 1
ore44
E-18/AF317694/Metcalf
E-18/JN203852/N-904B/IN
E-18/LT883145/TN
E-18/HM777023/Kor05-054cn/KR/2005
E-18/LN876167/CF159050 TH FR07
EV-B81/AY843299
EV-B87/AY843305
E-27/AY302551
EV-B97/AY843307
E-17/AY302543
EV-B88/AY843306
EV-B98/AB426608
ok135
se10
EV-C116/KT285370/M95/CI/2014
EV-C116/MH084298/Env SEN Feb2013
EV-C116/JX514942
CV-A1/AF499635
CV-A22/AF499643
EV-C113/KC344833
CV-A19/AF499641
EV-C104/EU840733
EV-C117/JX262382
EV-C118/JX393301
EV-C105/JX514943
EV-C109/GQ865517
CV-A24v/AB769154/Okinawa/7/JP/2011
CV-A24v/DQ443001
CV-A24v/AY876912/Ningbo2-02/CN/2002
CV-A24v/EF015040/USA-Fl98-10631
epc04
CV-A24/EF026081/Joseph
EV-C99/EF555644
EV-C99/EF015009
EV-C99/JX417882/DOU-064
EV-C99/KY748294/NGR AFP CN28a 2015
EV-C99/MG252514/CCN NGR 2015
ok82
CV-A21/AF465515
CV-A11/AF465512/G-9
EV-C96/EF015886
PV-2/AY184220
PV-3/AY184221
PV-1/AY184219
CV-A20/EF015019/Cecil
CV-A20/JQ744308/37-Ma-197/PHL
ore020
CV-A20/JN255675/CAF-LOB-05-077
CV-A20/AF499642/IH35
CV-A20/EF015021/Tulane
EV-C102/EF555645
CV-A17/AF499639
ore06 2
ore026
CV-A13/MG793389/03-1228/MW/2003
CV-A13/AF465511/Flores
CV-A13/JN255666/CAF-BAN-99-010
CV-A13/MF686568/AFP LN24 NGR 2015
CV-A13/JX417839/13 KOL-408
CV-A13/JX426648/TOK-377
ore043
0.2

Supplementary Material Table 2: Characteristics of patients with cosavirus positive samples and molecular results

Sample ID	Name of location	Type of Location	Age (Y)	Sex	Collection date	5'NCR seq
OK 132	Okeosun	Semi--urban	70	Male	Mar,2016	Cosavirus D
BS 018	Ede	Urban	15-25	Female	July,2016	Cosavirus A
IS 004	Ede	Urban	15-25	Male	July,2016	Cosavirus D
EPC 17	Ede	Urban	22	Female	Sep,2016	Cosavirus D
SE 03	Okeosun	Semi--urban	43	Male	Feb,2016	Cosavirus A
ORE 043	Ore	Rural	4	Female	May,2017	Cosavirus A
ORE 09	Ore	Rural	4	Female	May,2017	Cosavirus D
ORE 44	Ore	Rural	5	Female	May,2017	Cosavirus D
OK 84	Okeosun	Semi--urban	6	Male	Mar,2016	Cosavirus D
ORE 010	Ore	Rural	7	Female	May,2017	Cosavirus A
ORE 032	Ore	Rural	7	Male	May,2017	Cosavirus A
ORE 030	Ore	Rural	8	Male	May,2017	Cosavirus D
ORE 019	Ore	Rural	9	Female	May,2017	Cosavirus A
OK 68	Okeosun	Semi--urban	10	Female	Mar,2016	Cosavirus D
ORE 017	Ore	Rural	10	Female	May,2017	Cosavirus D
ORE 045	Ore	Rural	10	Female	May,2017	Cosavirus D
ORE 33	Ore	Rural	10	Male	May,2017	Cosavirus D
OK 78	Okeosun	Semi--urban	11	Female	Mar,2016	Cosavirus D
ORE 020	Ore	Rural	11	Female	May,2017	Cosavirus D
OK 72	Okeosun	Semi--urban	11	Male	Mar,2016	Cosavirus D
ORE 22	Ore	Rural	12	Female	May,2017	Cosavirus A
ORE BIO	Ore	Rural	12	Female	May,2017	Cosavirus D
ORE 035	Ore	Rural	14	Male	May,2017	Cosavirus D
ORE TAO	Ore	Rural	14	Male	May,2017	Cosavirus D
OK 71	Okeosun	Semi--urban	10	Male	Mar,2016	Cosavirus D
OK 155	Okeosun	Semi--urban	46	Male	Mar,2016	Cosavirus D
OK 35	Okeosun	Semi--urban	50	Female	Mar,2016	Cosavirus D
OK 42	Okeosun	Semi--urban	50	Female	Mar,2016	Cosavirus D
S 012	Ede	Urban	50	Male	Feb,2016	Cosavirus A
OK 59	Okeosun	Semi--urban	59	Female	Mar,2016	Cosavirus D
OK 146	Okeosun	Semi--urban	60	Female	Mar,2016	Cosavirus D
OK 116	Okeosun	Semi--urban	61	Female	Mar,2016	Cosavirus A
OK 153	Okeosun	Semi--urban	65	Female	Mar,2016	Cosavirus D

References:

1. Harvala, H. Epidemiology and clinical associations of human parechovirus respiratory infections Evaluation Studies. J Clin Microbiol. 2008, 46(10):3446-53. doi: 0.1128/JCM.01207-08. PMID: 18753351
2. Di Cristanziano, V.; Böttcher, S.; Diedrich, S.; Timmen-Wego, M.; Knops, E.; Lübke, N.; Kaiser, R.; Pfister, H.; Kaboré,Y.; D'Alfonso, R. Detection and characterization of enteroviruses and parechoviruses in healthy people living in the South of Côte d'Ivoire J Clin Virol 2015, 72:153. doi: 10.1016/j.jcv.2015.10.005.
3. Müller, B.; Klemm, U; Mas Marques, A; Schreier, E. Genetic diversity and recombination of murine noroviruses in immunocompromised mice. Arch Virol. 2007, 152(9):1709-19. DOI: 10.1007/s00705-007-0989-y
